# La_5_Ti_2_Cu_0.9_Ag_0.1_S_5_O_7_ Modified with a Molecular Ni Catalyst for Photoelectrochemical H_2_ Generation

**DOI:** 10.1002/chem.201801169

**Published:** 2018-06-06

**Authors:** Timothy E. Rosser, Takashi Hisatomi, Song Sun, Daniel Antón‐García, Tsutomu Minegishi, Erwin Reisner, Kazunari Domen

**Affiliations:** ^1^ Department of Chemical System Engineering Faculty of Engineering University of Tokyo 7-3-1 Hongo Bunkyo-ku Tokyo 113-8656 Japan; ^2^ Christian Doppler Laboratory for Sustainable SynGas Chemistry Department of Chemistry University of Cambridge Lensfield Road Cambridge CB2 1EW UK; ^3^ National Synchrotron Radiation Laboratory Collaborative Innovation Center of Chemistry for Energy Materials University of Science & Technology of China Hefei Anhui 230029 P. R. China; ^4^ Center for Energy & Environmental Science Shinshu University 4-17-1 Wakasato, Nagano-shi Nagano 380-8553 Japan; ^5^ Current affiliation: Center for Energy & Environmental Science Shinshu University 4-17-1 Wakasato, Nagano-shi Nagano 380-8553 Japan

**Keywords:** hydrogen, molecular electrochemistry, semiconductors, solar fuels, surface chemistry

## Abstract

The stable and efficient integration of molecular catalysts into *p*‐type semiconductor materials is a contemporary challenge in photoelectrochemical fuel synthesis. Here, we report the combination of a phosphonated molecular Ni catalyst with a TiO_2_‐coated La_5_Ti_2_Cu_0.9_Ag_0.1_S_5_O_7_ photocathode for visible light driven H_2_ production. This hybrid assembly provides a positive onset potential, large photocurrents, and high Faradaic yield for more than three hours. A decisive feature of the hybrid electrode is the TiO_2_ interlayer, which stabilizes the oxysulfide semiconductor and allows for robust attachment of the phosphonated molecular catalyst. This demonstration of an oxysulfide‐molecular catalyst photocathode provides a novel platform for integrating molecular catalysts into photocathodes and the large photovoltage of the presented system makes it ideal for pairing with photoanodes.

Splitting water into H_2_ and O_2_ with sunlight in a photoelectrochemical (PEC) cell holds great promise for sustainable fuel production.^1^, ^2^ However, the limited availability of high‐performance photocathodes that 1) utilize the long wavelengths of sunlight,^3^ 2) avoid photocorrosion in aqueous solution^4^, ^5^ and 3) do not require precious metal co‐catalysts^6^ remains a bottleneck for applications.

Oxysulfides La_5_Ti_2_CuS_5_O_7_ (LTC) and La_5_Ti_2_Cu_0.9_Ag_0.1_S_5_O_7_ (LTCA) have emerged as promising photocathode materials,^7^, ^8^, ^9^ with the latter absorbing light up until 710 nm and demonstrating PEC H_2_ production from aqueous solution, with an onset potential of 0.8 V versus the reversible hydrogen electrode (RHE) when using Pt as a co‐catalyst.^8^ Surface modification of LTC and LTCA photocathodes with thin TiO_2_ layers improves their activity due to enhanced charge separation,^10^, ^11^ and layers of amorphous TiO_2_ have been shown to protect LTCA under strongly alkaline (pH 13) conditions.^12^

Molecular complexes of Earth‐abundant metals are alternatives to precious metal nanoparticles, where tuning the primary and secondary coordination spheres allows obtaining very high per site activities.^13^ In addition, modification of the outer coordination shell of molecular catalysts allows for specific anchoring onto photocathode surfaces.^14^, ^15^ Phosphonic acid anchoring groups have a high affinity for metal oxide surfaces under acidic and pH neutral aqueous conditions,^16^, ^17^ and are thus well suited for immobilizing molecular catalysts to photocathodes stabilized with TiO_2_ protection layers.^18^

In this study, we report an LTCA photocathode that is stabilized by a sputtered TiO_2_ layer and modified with a molecular H_2_ evolution catalyst. As catalyst, we employ a Ni bis(diphosphine)‐based complex (**NiP**, Figure 1), which shows optimal activity under mildly acidic conditions,^19^ and has previously been employed on a mesoporous TiO_2_ electrode and a TiO_2_‐protected *p*‐Si photocathode.^20^, ^21^ The resulting LTCA|TiO_2_|**NiP** photoelectrode represents a new type of molecular‐semiconductor hybrid photocathode with high PEC performance and stability, as well as expanding the operating conditions of LTCA to acidic aqueous solution.

**Figure 1:**
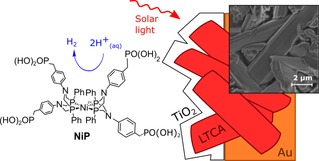
Schematic representation of solar‐light driven reduction of aqueous protons to H_2_ with the LTCA|TiO_2_|**NiP** photocathode. An SEM image of LTCA|TiO_2_|**NiP** is shown as inset.

Particulate LTCA was prepared as previously described,^8^, ^9^ and was assembled into freestanding photocathodes with an Au backing layer. Nanostructured LTCA films were subsequently modified with a thin (approx. 2 nm) layer of TiO_2_ by reactive RF magnetron sputtering, and annealed under air to form electrodes referred to herein as LTCA|TiO_2_ (see Supporting Information for details). Sputtering was used because this technique has previously been shown to increase the activity of LTC and LTCA photocathodes by minimizing charge recombination.^10^, ^11^ The TiO_2_ layer also stabilizes the LTCA under the otherwise corrosive acidic conditions required for **NiP** to operate,^19^, ^22^ as well as providing a proven attachment site for this catalyst.^20^, ^21^ Scanning electron microscopy (SEM, Figure 1) of these electrodes shows the rod‐like structure of the LTCA particles with lengths of approximately a few micrometers.

Immobilization of **NiP** on LTCA|TiO_2_ electrodes was achieved by submersion in a methanol solution (0.5 mm) overnight. The presence of **NiP** was confirmed by the observation of N, P, and Ni in the XPS spectrum of the modified electrode (Figure S1 in Supporting Information). The **NiP** surface loading was quantified as 33.7±2.4 nmol cm^−2^ by desorption from LTCA|TiO_2_ in aqueous NaOH solution (0.1 m), followed by UV/Vis spectroscopic analysis (see Supporting Information).^20^ This value is in the expected range for a mesostructured electrode modified with a phosphonated metal complex.^17^, ^21^

The PEC properties of the LTCA|TiO_2_|**NiP** electrode were first studied in aqueous Na_2_SO_4_ solution at pH 3 under simulated solar irradiation (AM 1.5G, 100 mW cm^−2^). The linear sweep voltammetry (LSV) scans shown in Figure 2 a show a cathodic onset photocurrent at approximately 0.65 V versus RHE with a photocurrent of −0.6 mA cm^−2^ at 0 V versus RHE. Note that the LSV response does not represent an improvement compared to the LTCA|TiO_2_ electrodes without catalyst modification on this timescale (approximately 1 min). This may be due to a photoreductive decomposition process occurring for LTCA|TiO_2_, leading to initially higher but short‐lived catalytic activity (see below).

**Figure 2:**
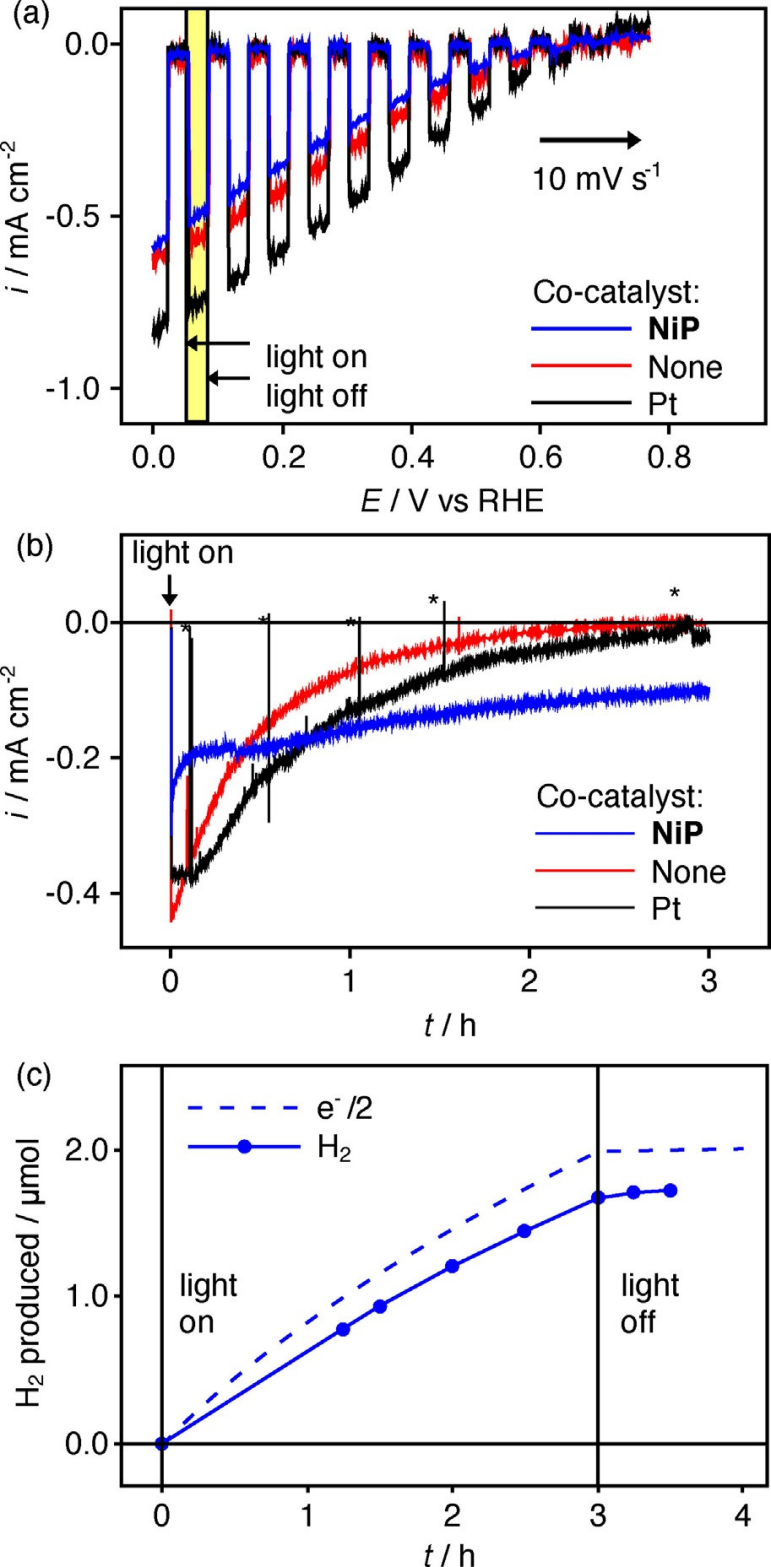
a) Light chopped LSV (scan rate=10 mV s^−1^, anodic direction) and b) CPPE at +0.3 V vs. RHE of LTCA|TiO_2_ photocathodes modified with **NiP**, no co‐catalyst or Pt (* indicates where irradiation was temporarily interrupted in CPPE) c) H_2_ quantification for LTCA|TiO_2_|**NiP** from (b). The dashed line represents the theoretical amount of H_2_ based on the charge passed (i.e., 100 % Faradaic yield). Conditions: Aqueous Na_2_SO_4_ (0.1 m) at pH 3 electrolyte solution under simulated solar irradiation (AM1.5G) and a constant Ar purge.

Nevertheless, controlled potential photoelectrolysis (CPPE, Figure 2 b) during irradiation at +0.3 V versus RHE reveals a different response between **NiP**‐modified and bare LTCA|TiO_2_ electrodes. The latter shows an initial photocurrent of −0.4 mA cm^−2^, which decays quickly to zero within three hours, indicating instability and/or lack of catalysis. In contrast, prolonged CPPE with LTCA|TiO_2_|**NiP** under the same conditions showed an initial photocurrent of −0.2 mA cm^−2^, which was retained at 50 % after 3 hours irradiation (Figure 2 b). Product quantification through gas chromatography during a CPPE experiment with LTCA|TiO_2_|**NiP** reveals that 1.7 μmol H_2_ was produced, representing a turnover number (TON) of approximately 50 per initial Ni site. The Faradaic yield was measured to be 87 % (Figure 2 c), which matches the values obtained for previously reported **NiP** catalysts anchored on TiO_2_ cathodes^20^ and photocathodes.^21^ Longer term CPPE (Figure S2 in Supporting Information) demonstrated continued photocurrent for at least 6 h, although with a steadily declining activity that is consistent with catalyst desorption or degradation. In the absence of **NiP**, H_2_ production had essentially ceased after 2 h (Figure S3 a), suggesting that this is accompanied by a degradation process that is avoided when the electrons are harvested by the **NiP** catalyst. It has previously been shown that **NiP** can efficiently accept electrons from the TiO_2_ conduction band,^19^, ^20^ which slows the otherwise rapid degradation of the LTCA semiconductor material.

The LTCA|TiO_2_|**NiP** photocathodes were further characterized by measuring the single wavelength incident photon‐to‐current efficiencies (IPCE) across the visible spectrum with an applied potential of +0.3 V versus RHE. The photocathodes demonstrated photocathodic current at wavelengths as long as 660 nm, matching the diffuse reflectance UV/Vis spectrum (Figure 3). An IPCE of 2.4 % was recorded at 440 nm.

**Figure 3:**
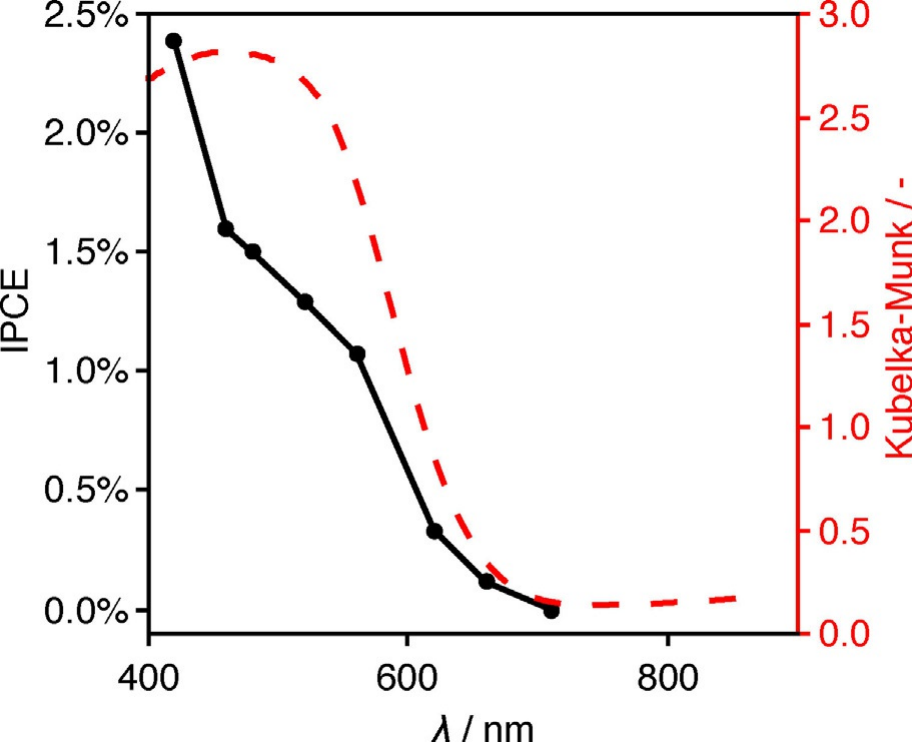
Black line: Incident photon‐to‐current efficiency (IPCE) spectrum of LTCA|TiO_2_|**NiP** photocathode in pH 3 Na_2_SO_4_ (0.1 m) electrolyte solution irradiated with a 300 W Xe lamp fitted with narrow band filters under a purge of Ar and an applied potential of 0.3 V vs. RHE. Red dashed line: DRS UV/Vis spectrum of LTCA powder (in Kubelka–Munk absorbance units).

Analysis of immobilized **NiP** on the electrode surface after a three‐hour CPPE experiment (quantified by UV/Vis spectroscopy following desorption in aqueous NaOH, see above) revealed a surface loading of 16 nmol cm^−2^. This represents a loss of approximately half of the initial catalyst from the electrode surface during CPPE and matches the 50 % drop in PEC activity (Figure 2 b). Moreover, a qualitative resemblance between the UV/Vis peaks of the desorbed **NiP** before and after CPPE (Figure S4), as well as retention of peaks corresponding to N and P in the XPS spectra after 1 h CPPE (Figure S1), support the molecular integrity of **NiP** during the PEC experiments. Ni signals belonging to either **NiP** or a decomposition product could not be clearly resolved in the post‐CPPE XPS spectra.

Control experiments were performed without a sputtered TiO_2_ layer. A **NiP** loading of 17.6±3.5 nmol cm^−2^ was determined for LTCA|**NiP**, which is approximately half that observed for LTCA|TiO_2_|**NiP**, and thus in agreement with the particular affinity between the phosphonate‐modified molecules and TiO_2_.^15^, ^23^ Moreover, when these electrodes were subjected to CPPE at +0.3 V versus RHE under the same conditions as described above (Figure S5), the initially low photocurrent of −0.15 mA cm^−2^ approached zero within two hours, demonstrating the requirement for a stabilizing layer. Thus, TiO_2_ adds two further benefits to the increase in photocurrent observed at pH 10^10^, ^11^—it stabilizes LTCA and provides an improved anchoring site for **NiP**.

LTCA|TiO_2_ electrodes modified with Pt instead of **NiP** were prepared by PEC reduction of H_2_PtCl_6_ (see Supporting Information for details). When studied in pH 10 electrolyte solution, the most commonly used conditions for these materials,^8^ the electrodes displayed a photocurrent of −1.0 mA cm^−2^ at 0 V versus RHE in the LSV and relatively stable H_2_ production for two hours at *E*_appl_=+0.5 V versus RHE (Figure S6). In pH 3 electrolyte solution, the LSV of the LTCA|TiO_2_|Pt electrodes displayed a similar onset potential to LTCA|TiO_2_|**NiP** electrodes with photocurrent reaching −0.8 mA cm^−2^ at 0 V versus RHE (Figure 2 a). However, CPPE at +0.3 V versus RHE showed a much quicker decay than with the LTCA|TiO_2_|**NiP** electrodes, falling to only −0.01 mA cm^−2^ after three hours irradiation and a Faradaic yield of 84 % (Figure 2 b and Figure S3 b). This decay is consistent with previous studies, where the contact between this TiO_2_ protecting layer and the Pt catalyst has limited the durability of the photocathode,^24^ and either an additional Mo/Ti layer^25^ or replacing the Pt co‐catalyst with a thick RuO_*x*_ film^26^ has been required to achieve long term performance. Our photoelectrode therefore provides a rare example where the chemical attachment between a molecular catalyst and the electrode is improved compared to a precious metal catalyst layer, and indeed the molecular catalyst appears essential for operation of the LTCA material under mildly acidic conditions. H_2_ production by LTC or LTCA from acidic solution has not previously been demonstrated, and therefore the use of both the TiO_2_ stabilizing layer and molecular catalyst expands the possible operating conditions of this class of material.

This work represents an advance in the assembly of hybrid semiconductor/molecular catalyst photocathodes due to its positive operating potential and long wavelength activity. The onset potential of +0.65 V versus RHE is significantly more positive than for *p*‐Si modified with TiO_2_ and the same **NiP** co‐catalyst,^21^ and is comparable to the values reported using GaP‐based hybrid photocathodes.^27^, ^28^ Additionally, our LTCA hybrid photocathodes allow H_2_ production up to *λ*=660 nm, which exceeds that of GaP, where the band gap limits light absorption to 549 nm.^14^ The use of the particle transfer fabrication method, compared to flat semiconductor wafers such as *p*‐Si and GaP, makes this electrode scaffold potentially scalable over large areas. The excellent contact between the particles and the contact layer (Au in this case) removes the requirement for high crystallinity across the whole panel,^29^, ^30^ and the intrinsically mesostructured surface enables high catalyst loading. However, the loss of activity is somewhat faster than for the previous (photo)cathodes using the same catalyst.^20^, ^21^ Previous systems utilized mesoporous TiO_2_ with several‐μm‐thick films, which trap the catalyst through re‐adsorption following desorption, when compared to the nm‐thick TiO_2_ layer reported here. Therefore, increasing the porosity of the TiO_2_ layer may improve the system's longevity in future development. Finally, LTCA|TiO_2_|**NiP** compares favourably in terms of photocurrent and Faradaic yield with “all‐molecular” dye/H_2_‐catalyst assemblies, which are typically immobilised on *p*‐type materials such as NiO and CuCrO_2._ 31, 32, 33, 34, 35

In conclusion, photocathodes based on the oxysulfide La_5_Ti_2_Cu_0.9_Ag_0.1_S_5_O_7_ were combined with a molecular catalyst, enabling early‐onset H_2_ fuel synthesis with a molecular/inorganic hybrid. This was accomplished by employing a sputtered TiO_2_ layer, which protects the material from the aqueous electrolyte solution and provides a suitable attachment site for the phosphonic acid‐modified molecular catalyst **NiP**. The latter was crucial for the high performance of the photocathode, which still retained 50 % of its initial activity after three hours photoelectrolysis. An analogous photocathode modified with Pt displayed poor stability, giving a rare example where a molecular catalyst exceeds the activity and stability of Pt for H_2_ production.^36^ Our results demonstrate the possibility of replacing expensive Pt with a first row transition metal catalyst for oxysulfide‐type photocathode materials. The use of a TiO_2_ overlayer opens up their use in previously unreported acidic conditions that may be required in tandem PEC device architectures. From a molecular catalysis point of view, this is the most positive operating potential for an inorganic light‐harvester/molecular catalyst hybrid, and the system retains high Faradaic efficiency even at this potential. This is therefore an essential step towards constructing an efficient, bias‐free molecule‐catalysed photoelectrochemical cell.

## Experimental Section

Experimental details can be found in the Supporting Information.

## Conflict of interest

The authors declare no conflict of interest.

## Supporting information

As a service to our authors and readers, this journal provides supporting information supplied by the authors. Such materials are peer reviewed and may be re‐organized for online delivery, but are not copy‐edited or typeset. Technical support issues arising from supporting information (other than missing files) should be addressed to the authors.

Supplementary
