# Supplementary material for: La5Ti2Cu0.9Ag0.1S5O7 Modified with a Molecular Ni Catalyst for Photoelectrochemical H2 Generation
Source: Chemistry. 2018 Jun 6;24(69):18393–7. doi: 10.1002/chem.201801169 (PMC6348378; doi:10.1002/chem.201801169)
Supplement: Supplementary file 1 — Supplementary [file CHEM-24-18393-s001.pdf]

CHEMISTRY
A European Journal

Supporting Information

$La_5Ti_2Cu_{0.9}Ag_{0.1}S_5O_7$ Modified with a Molecular Ni Catalyst for Photoelectrochemical H_2 Generation

Timothy E. Rosser,[a, b] Takashi Hisatomi,[a, e] Song Sun,[a, c] Daniel Antón-García,[b] Tsutomu Minegishi,[a] Erwin Reisner,*[b] and Kazunari Domen*[a, d]

chem_201801169_sm_miscellaneous_information.pdf

Materials and electrode preparation

Gallium-doped (1%) $La_5Ti_2Cu_{0.9}Ag_{0.1}S_5O_7$ (LTCA)[1,2] and Ni(bis-diphosphine) molecular catalyst ([Ni($P_2^{Ph}N_2^{PhCH_2PO_3H_2}$)$_2$]Br_2·HBr, $P_2^{Ph}N_2^{PhCH_2PO_3H_2}$ = (1,5-phenyl-diphospha-3,7-4-(phosphonmethyl)phenyl-diazacyclooctane); **NiP)**[3] were prepared according to published procedures. All other materials were obtained from commercial sources and used without further purification.

Photocathodes were prepared by the particle transfer method.[4] Firstly, 50 mg LTCA was suspended in 1 mL 2-propanol by ultrasonication for 30 mins, and drop cast onto 4 slides each measuring 3 x 1 cm. After drying, Au was evaporated onto the LTCA by vacuum evaporation to a thickness of approximately 3 μm. The Au|LTCA composite was peeled away from the glass substrate, and excess particles removed by gentle ultrasonication between two glass plates.

The TiO_2 layer was deposited by reactive RF (70 W) magnetron sputtering[5] with a high-purity Ti target and O_2 and Ar partial pressures of 0.05 and 0.07 Pa, respectively, at room temperature, for 240 s unless otherwise stated. This method was previously reported as yielding a deposition rate of approximately 0.5 nm min^{-1} using the same instrument under the same conditions.[2] The Au|LTCA|TiO_2 composites were subsequently annealed at 200 °C for 1 h in air. The Au|LTCA control electrodes were also annealed under the same conditions.

Modification with **NiP** was achieved by submersion of the free-standing Au|LTCA|TiO_2 composites in a 0.5 mM solution of **NiP** in methanol overnight, followed by brief submersion in neat MeOH to remove non-chemisorbed **NiP** and drying by evaporation in air. Subsequently, the **NiP**-modified electrodes were supported on glass plates with backing carbon tape and a wire attached to the Au backing layer with In solder. Finally, the non-LTCA areas of the electrode were covered with epoxy resin leaving no conducting areas exposed.

Quantification of **NiP** was achieved by submersion of the modified electrodes in 3 mL aqueous NaOH (0.1 M) for 5 min, followed by removal of the electrode and UV/vis spectroscopy of the resultant solution. Concentrations were obtained by interpolation of the absorbance at λ = 255 nm on a calibration curve compiled from known concentrations of fresh **NiP** dissolved in 0.1 M NaOH.

Where required, modification with Pt was achieved by first mounting the Au|LTCA|TiO_2 composite onto glass with carbon tape, attaching a wire with In and sealing with epoxy resin as described above. The resultant electrode was subsequently placed in a pH 10 solution of Na_2SO_4, $Na_2C_2O_4$ (both 0.1 M), and H_2PtCl_6 (3.5 μM) and held at a potential of 0.5 V vs RHE under simulated solar irradiation until the photocurrent stopped increasing (approximately 1 h). The electrode was finally rinsed with deionised water and dried under a stream of N_2.

UV/vis absorbance and diffuse reflectance spectra were obtained using a Jasco V-670 spectrophotometer.

XPS spectra were obtained on a JPS-90MX spectrometer using an Mg Kα source (8 kV, 10 mA) and a pass energy of 10 eV.

Electrochemical methods

All electrochemical measurements were carried out on a Hokutodenko HSV-110 potentiostat with a working electrode as described above, a Ag/AgCl/KCl(sat) reference electrode (RE) and a Pt wire counter electrode (CE), under an Ar atmosphere and with stirring. All linear sweet voltammetry (LSV) and controlled potential photoelectrolysis (CPPE) experiments were performed at room temperature

and after purging the electrolyte solution with Ar for 20 min and the solutions were kept under a constant purge throughout the measurements. LSV scans were performed with a scan rate of 10 mV s^{-1} starting at the most negative potential (0 V vs RHE). Electrochemical measurements were typically performed in 0.1 M Na_2SO_4, adjusted to the required pH (as stated) by addition of H_2SO_4 or NaOH solutions, unless otherwise stated.

Post-CPPE XPS spectra were obtained from electrodes previously subjected to 1 h CPPE in pH 3 Na_2SO_4 solution (0.1 M) at 0.3 V vs RHE, followed by rinsing with H_2O and drying under a stream for N_2.

Simulated solar irradiation was produced by a SAN-EI Electric Co. Ltd XES-40S2-CE solar simulator with an AM1.5G filter (λ > 300 nm) and measurements were performed at a position calibrated to 1 sun intensity.

IPCE measurements were performed at an applied potential of 0.3 V vs RHE in pH 3 Na_2SO_4 electrolyte solution. Single wavelength illumination obtained from an Asahi Spectra MAX-302 300 W Xe lamp equipped with narrow band-pass filters of the required wavelengths was applied for 30 s at each wavelength and the current density recorded, before ceasing the illumination for 10 s and applying the next wavelength. Light intensities were recorded with a calibrated Si photodiode (S2281-01, Hamamatsu Photonics).

H_2 evolution measurements were performed in a sealed electrochemical cell with 84 mL of 0.1 M Na_2SO_4 (adjusted to pH 3) used as the electrolyte solution. The cell was purged with Ar for 1 h prior to measurement with the electrodes (modified LTCA working electrode, Pt wire CE and Ag/AgCl RE) submerged in the electrolyte solution, after which the cell was sealed and a potential of 0.3 V vs RHE and simulated solar irradiation were applied for 3 h. The headspace was sampled periodically and analysed using an Agilent 490 microGC. After 3 h, the potential was applied for a further hour without irradiation and the headspace was sampled for GC analysis until a plateau in H_2 was reached. Calibration of H_2 was achieved by electrolysis of the same electrolyte solution with a Pt anode and cathode.

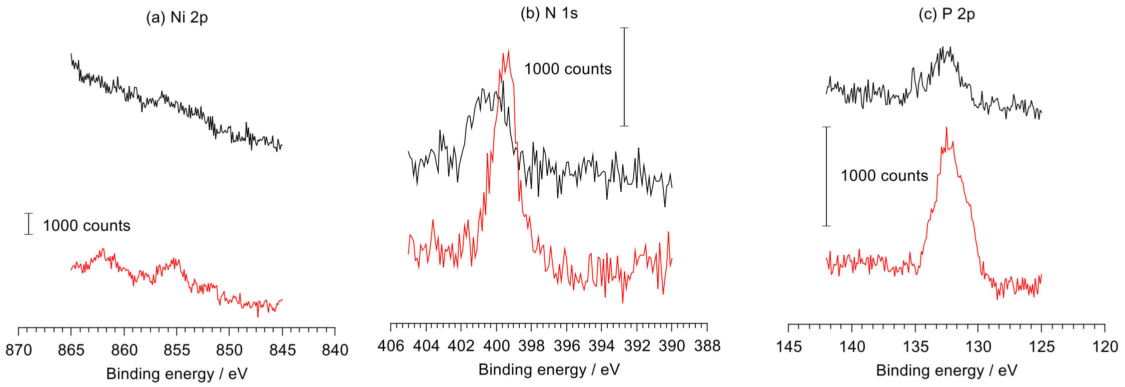

Figure S1. XPS spectra of LTCA|TiO_2|**NiP** as prepared (red line) and after CPPE for 1 h (black line) in the (a) Ni 2p, (b) N 1s and (c) P 2p regions. The broadening of the N signal in the post CPPE spectrum in (b) could be explained by protonation of the amine ligand, the binding energy of which occurs at slightly higher values,[6] after exposure to the acidic conditions of the CPPE experiment.

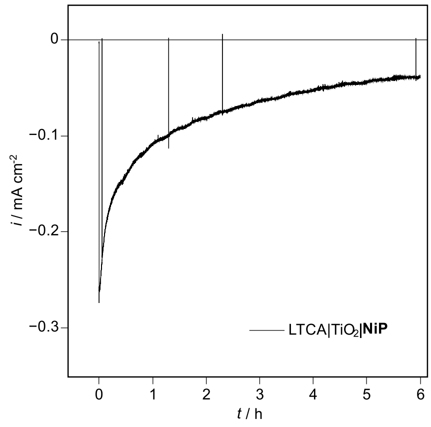

Figure S2 Longer-term (6 h) CPPE for an LTCA|TiO_2|**NiP** electrode at E_{appl} = 0.3 V vs RHE performed in aqueous Na_2SO_4 (0.1 M) electrolyte solution (pH 3) under simulated solar irradiation (AM1.5G) under a constant Ar purge at room temperature.

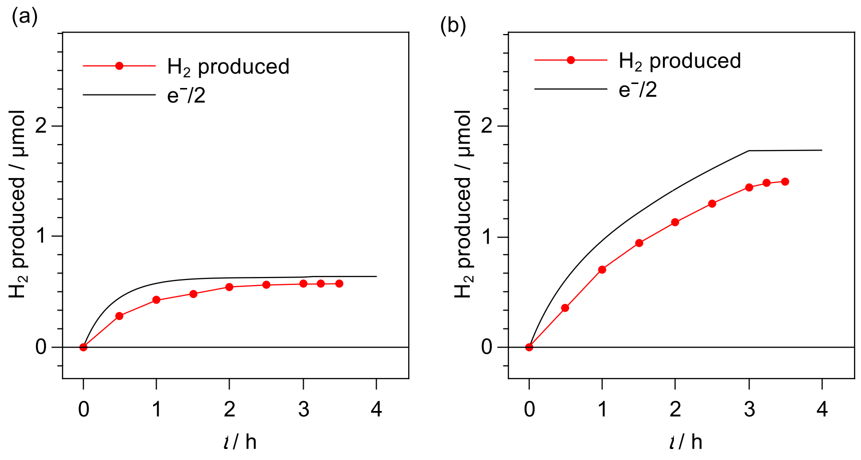

Figure S3. H_2 evolution on (a) LTCA|TiO_2 and (b) LTCA|TiO_2|Pt electrodes in pH 3 Na_2SO_4 (0.1 M) at E_{appl} = 0.3 V vs RHE under simulated solar irradiation (AM1.5G) under an Ar atmosphere. (Values are normalised to an electrode area of 1 cm^2). Experimental data are plotted in red and theoretical H_2 yields based on quantitative Faradaic yield in black.

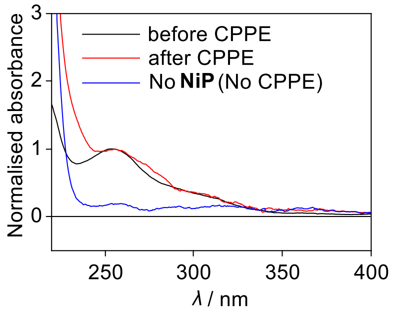

Figure S4. UV/vis spectra of **NiP** desorbed from the surface of LTCA|TiO_2|**NiP** and LTCA|TiO_2 electrodes before and after CPPE at E_{appl} = 0.3 V vs RHE in pH 3 solution under simulated solar irradiation (AM1.5G) for 3 h.

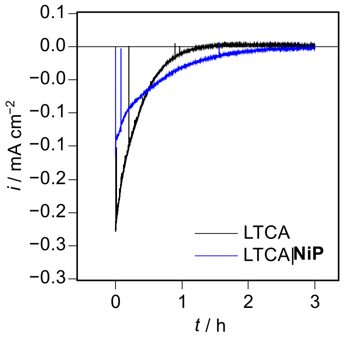

Figure S5. CPPE of LTCA photocathodes (no TiO_2) modified with **NiP** with E_{appl} =0.3 V vs RHE performed in Na_2SO_4 (0.1 M) electrolyte solution (pH 3) under simulated solar irradiation (AM1.5G) under a constant Ar purge.

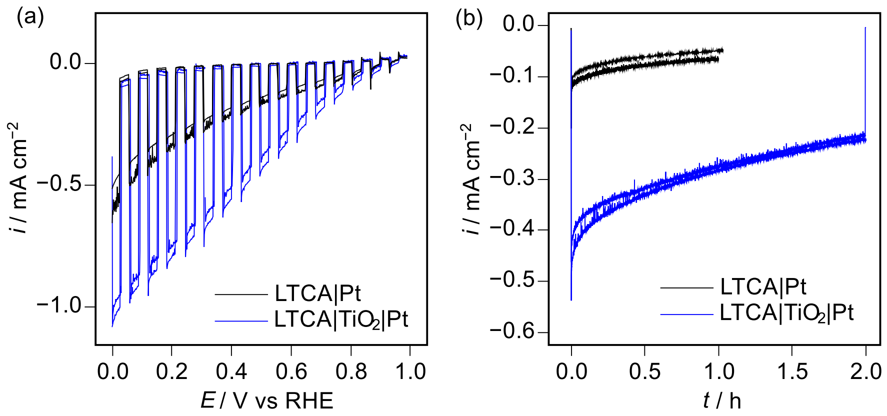

Figure S6. (a) Linear sweep voltammetry scans (ν = 10 mV s^{-1}) and (b) CPPE at E_{appl} = 0.5 V vs RHE of LTCA and LTCA|TiO_2 electrodes modified with Pt (by photoelectrochemical deposition) studied in pH 10 Na_2SO_4 (0.1 M) solution under simulated solar irradiation under a constant purge of Ar.

Supporting references

1 T. Hisatomi, S. Okamura, J. Liu, Y. Shinohara, K. Ueda, T. Higashi, M. Katayama, T. Minegishi and K. Domen, *Energy Environ. Sci.*, 2015, **8**, 3354–3362.

2 J. Liu, T. Hisatomi, M. Katayama, T. Minegishi and K. Domen, *ChemPhotoChem*, 2018, **2**, 234–239.

3 M. A. Gross, A. Reynal, J. R. Durrant and E. Reisner, *J. Am. Chem. Soc.*, 2014, **136**, 356–366.

4 J. Liu, T. Hisatomi, G. Ma, A. Iwanaga, T. Minegishi, Y. Moriya, M. Katayama, J. Kubota and K. Domen, *Energy Environ. Sci.*, 2014, **7**, 2239–2242.

5 J. Liu, T. Hisatomi, D. H. K. Murthy, M. Zhong, M. Nakabayashi, T. Higashi, Y. Suzuki, H. Matsuzaki, K. Seki, A. Furube, N. Shibata, M. Katayama, T. Minegishi and K. Domen, *J. Phys. Chem. Lett.*, 2017, **8**, 375–379.

6 NIST X-ray Photoelectron Spectroscopy Database, Version 4.1 (National Institute of Standards and Technology, Gaithersburg, 2012), http://srdata.nist.gov/xps/.

End of Supporting Information
